# Effects of Electroacupuncture on Methamphetamine-Induced Behavioral Changes in Mice

**DOI:** 10.1155/2017/5642708

**Published:** 2017-03-16

**Authors:** Tsung-Jung Ho, Chiang-Wen Lee, Zi-Yun Lu, Hsien-Yuan Lane, Ming-Horng Tsai, Ing-Kang Ho, Chieh-Liang Huang, Yao-Chang Chiang

**Affiliations:** ^1^Center for Drug Abuse and Addiction, China Medical University Hospital, China Medical University, Taichung, Taiwan; ^2^School of Chinese Medicine, China Medical University, Taichung, Taiwan; ^3^Division of Chinese Medicine, China Medical University Beigang Hospital, Yunlin County, Taiwan; ^4^Division of Chinese Medicine, An Nan Hospital, China Medical University, Tainan, Taiwan; ^5^Division of Basic Medical Sciences, Department of Nursing, Chang Gung Institute of Technology and Chronic Diseases and Health Promotion Research Center, Chiayi, Taiwan; ^6^Research Center for Industry of Human Ecology, Chang Gung University of Science and Technology, Kweishan, Taoyuan, Taiwan; ^7^Research Center for Chinese Medicine & Acupuncture, China Medical University, Taichung, Taiwan; ^8^Graduate Institute of Biomedical Sciences and Ph.D. Program for Aging, China Medical University, Taichung, Taiwan; ^9^Department of Psychiatry, China Medical University Hospital, Taichung, Taiwan; ^10^Department of Pediatrics, Division of Neonatology and Pediatric Hematology/Oncology, Chang Gung Memorial Hospital, Yunlin, Taiwan

## Abstract

Methamphetamine (METH) is a major drug of abuse worldwide, and no efficient therapeutic strategies for treating METH addiction are currently available. Continuous METH use can cause behavioral upregulation or psychosis. The dopaminergic pathways, particularly the neural circuitry from the ventral tegmental area to the nucleus accumbens (NAc), have a critical role in this behavioral stage. Acupuncture has been used for treating diseases in China for more than 2000 years. According to a World Health Organization report, acupuncture can be used to treat several functional disorders, including substance abuse. In addition, acupuncture is effective against opioids addiction. In this study, we used electroacupuncture (EA) for treating METH-induced behavioral changes and investigated the possible therapeutic mechanism. Results showed that EA at the unilateral* Zhubin* (KI9)–*Taichong* (LR3) significantly reduced METH-induced behavioral sensitization and conditioned place preference. In addition, both dopamine and tyrosine hydroxylase (TH) levels decreased but monoamine oxidase A (MAO-A) levels increased in the NAc of the METH-treated mice receiving EA compared with those not receiving EA. EA may be a useful nonpharmacological approach for treating METH-induced behavioral changes, probably because it reduces the METH-induced TH expression and dopamine levels and raises MAO-A expression in the NAc.

## 1. Introduction

Drug abuse is a serious social, economic, and health concern worldwide. Methamphetamine (METH) is a commonly abused drug. Chronic exposure to METH induces behavioral sensitization—the progressive enhancement of behavioral response during repetitive drug administration—which persists even after long withdrawal periods [1, 2]. METH-induced behavioral sensitization is associated with the activation of mesolimbic and mesocortical dopaminergic system—a series of neurochemical and neurophysiological changes occur in the ventral tegmental area with time, leading to long-term structural and neurochemical alterations in the nucleus accumbens (NAc), an area critical for behavioral expression [3]. Dopamine (or 3,4-dihydroxyphenethylamine) levels in the NAc have a critical role in addiction development [4]. Dopamine levels increase after acute administration or chronic METH-pretreated mice reexposure to METH, both of which increase tyrosine hydroxylase (TH) expression [4]. In addition, levels and activities of the monoamine metabolic enzyme-monoamine oxidases (MAOs) are also associated with METH-induced behavioral and cellular changes [5]. According to a 2014 report from the United States [6], METH use was associated with 102,961 emergency room visits in 2011; the number of such visits increased 1.5-fold from 2007 to 2011, which is indicative of the increasing numbers of METH addicts. Thus, developing methods for reducing and treating METH abuse is imperative.

For more than 2000 years, acupuncture, a traditional medical technique, has been used for treating diseases in China. In 1979, the World Health Organization (WHO) provided a list of 40 diseases suitable for acupuncture treatment; these included heroin and cocaine dependence and abuse [7]. Some clinical and basic studies have suggested that acupuncture, as a therapeutic tool, is useful for reducing drug abuse-induced seeking behaviors and side effects by restoring cellular function imbalance in the central nervous system (CNS) [8–10]. Acupuncture at several specific acupoints could reduce cocaine-induced seizures, behavioral sensitization, stress-induced cocaine relapse, and METH-induced hyperactivity in rodent models [8–10]. In addition, clinical studies have validated that electroacupuncture (EA) at* Hegu* (LI4) and* Zusanli* (ST36) can improve sleep latency in methadone maintenance therapeutic patients [11] and that three auricular sites and one body site on LI4 significantly decreases cocaine use in methadone-treated patients [12]. Several studies have suggested that acupuncture ameliorates the effects of drug abuse. However, some studies have provided contradictory results [13, 14]: therapeutic effects of acupuncture on drug addiction are unclear, and the underlying mechanisms require further investigation [7, 15–17].

Unlike methods for opioid addiction, which is treatable through the methadone or buprenorphine maintenance program, effective methods for treating psychostimulants addiction are yet to be developed. The present study evaluated the possible therapeutic effects of EA on a chronic METH-treated murine model. Our results suggested that EA intervention reduces chronic METH-induced behavioral changes probably by regulating dopamine release and synthesis.

## 2. Methods

### 2.1. Animals

**Figure 1:**
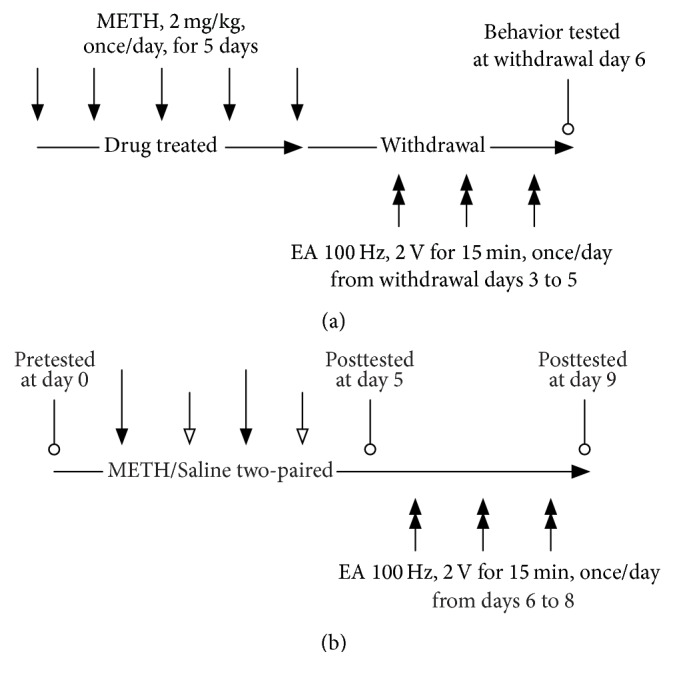
Treatment schedule for electroacupuncture and METH administration in mice. (a) Behavioral sensitization. (b) Conditioned place preference.

Male C57BL/6 mice (National Laboratory Animal Center, Taipei, Taiwan), weighing 20–30 g, were acclimatized to a room maintained at 25°C and 50%  ± 10% humidity under a 12 h day-night cycle (lights on 08:00–20:00 h) for at least 3 days before experimentation. The mice were housed 4-5 per cage and were provided with food (LabDiet 5053, PMI Nutrition International, St. Louis, MO, USA) and water ad libitum. All behavioral tests were performed on mice aged 8–12 weeks. The test schedule is detailed in Figure 1. Ethical guidelines established by the China Medical University Animal Core (IACUC number: 102-238-C) were followed throughout the study.

### 2.2. Drugs

Methamphetamine HCL (Factory for Controlled Drugs, Food and Drug Administration, Ministry of Health and Welfare, Taipei, Taiwan) was dissolved in saline (distilled 0.9% NaCl) and administered intraperitoneally (*i.p.*) at 10 mL/kg body weight. Other chemicals were purchased from Sigma-Aldrich (St. Louis, MO, USA).

### 2.3. Locomotor Activity Test

To examine the locomotor activity at the exploratory stage and behavioral sensitization induced by METH, all groups of mice were removed from their home cages and placed into a locomotor testing box (45 × 45 × 30 cm) for 30 min to measure their basal locomotor activities. Thereafter, mice were given 2 mg/kg* i.p.* of METH for 5 days to induce hyperlocomotor activities for 1 h. To examine behavioral sensitization, METH-treated group mice were regiven 1 mg/kg* i.p.* METH on withdrawal day 6 and their locomotor activities were recorded for 1 h. The monitoring and recording (300 ms for tracing time intervals) of locomotor activities were performed in an acoustically insulated room by using a video tracer software (Trace Mouse II, SINGA, Taiwan). Water was used to clean the inner surface of the apparatus. All experiments were performed during the light phase (08:00–20:00 h).

### 2.4. Conditioned Place Preference Test

For conditioned place preference (CPP) tests, the apparatus comprised an acrylic plastic box with two connected equal-sized compartments (15 × 15 × 25 cm). The four inner walls and floor were black in one compartment and white in the other; the walls and floor in both compartments were used for visual cues. First, the mice were randomly placed into one of the compartments and were given free access to the entire box for 15 min to determine their predrug place preferences. During conditioning, METH (2 mg/kg,* i.p.*) was paired with the nonpreferred white compartment, whereas the vehicle (saline) was paired with the black compartment. The mice were then kept in the compartment for 1 h in a corresponding compartment with the connecting doors closed. Two drug-paired and two vehicle-paired conditioning trials were performed before postdrug tests. The postdrug place preference was examined at days 5 and 9 for 15 min. Animal behaviors were recorded using the video tracer software.

### 2.5. EA

EA was repeated for 3 days—withdrawal days 3–5 and 6–8 for the behavioral sensitization and CPP tests, respectively. During EA stimulation period, animals were subjected to anesthesia with isoflurane inhalation with no pain response. Electrical stimulator (HC-0502, Home Care Technology, Co., Ltd., Tainan, Taiwan) provided 5 ms pulses at 100 Hz for 15 min following a previous study [9]. The current intensity was adjusted to evoke light muscle trembles (approximately 1.5–2 mV). Acupoint positions were based on the transposition model; in other words, the locations of acupoints on the animal corresponded to the anatomic sites of human acupoints [18, 19]. We examined three pairs of acupoints ipsilateral to avoid the electric current cross the heart:* Zusanli* (ST36)–*Taichong* (LR3),* Neiguan* (PC6)–*Quchi* (LI11), and* Zhubin* (KI9)–*Taichong* (LR3). Stainless-steel needles (36 G × 1/2′′ (.20 × 13 mm), Shanghai Yanglong Medical Articles Co., Ltd., Shanghai, China) were inserted 3-4 mm deep (1 mm depth for LR3) into the murine-equivalent human acupoints. In the sham group, a nonacupoint region (buttock) and a region 0.5 cm from LR3 or LI11 were selected and provided with electrical stimulus evoking no muscle trembles. ST36, PC6, and KI9 were with negative charge, and LR3, LI11, and buttock were with positive charge.

### 2.6. High-Performance Liquid Chromatography-Electrochemical Detection Assay

The NAc region was dissected from fresh brains and frozen in liquid nitrogen until use. NAc tissues were extracted with 0.1 N HClO_4_ and centrifuged at 19100 ×g for 30 min; the supernatant was then analyzed for catecholamines and amino acid transmitters by using a high-performance liquid chromatography (HPLC) system. The pellets were resuspended in 0.2 N NaOH to measure the protein concentration. The HPLC system (1260 infinity, Agilent Technologies, Santa Clara, CA, USA) included a reverse-phase C-18 column (150 × 4.6 mm internal diameter [*i.d.*], ODS, 5 mm particle size, Nucleosil and Nucleodur, Duren, Germany), a guard column (10 × 4.6 mm* i.d.*, ODS, Nucleosil and Nucleodur), 1260 quant pump VL (Agilent Technologies), 35900E interface (Agilent Technologies), and an electrochemical detector (CLC100, Chromsystems, Grafelfing, Bavaria, Germany). The electrochemical detector comprised a glassy carbon working electrode (Teflon cell gasket; 0.002) and an Ag/AgCl reference electrode. The applied oxidation potential was set at +0.65 V. The concentrations of dopamine, 3,4-dihydroxyphenylacetic acid, and homovanillic acid (standard compounds purchased from Sigma-Aldrich) were determined on the electrochemical detector with a mobile phase comprising 5 mM NaH_2_PO_4_, 30 mM citric acid, 0.1 mM EDTA, 0.02% sodium octylsulfate, and 9% methanol (pH 4.4). To quantify the sample peaks, each chemical was normalized to its protein concentration and then compared with the external standards, prepared freshly and injected in every ten sample runs.

### 2.7. Immunoblotting Analysis

The brain tissues were sonicated on ice in 1x RIPA buffer with proteinase inhibitors (Protease Inhibitor Cocktail Set III, Animal-Free-Calbiochem, Millipore, Billerica, MA, USA) three times for 5 s each. Small aliquots of the extracts were retained for protein determination by using bicinchoninic acid assay (Thermo scientific, Waltham, MA, USA), with bovine serum albumin as the standard. Thereafter, equal amounts of protein (20–40 *μ*g) were separated through SDS-polyacrylamide gel electrophoresis (10%–12.5% polyacrylamide) and transferred onto a polyvinylidene fluoride (Millipore) membrane; the signals were measured using an enhanced chemiluminescence detection kit (Millipore). The antibodies against TH (ab6211, Abcam, Cambridge, UK), TH-phosphoSer40 (AB5935, Millipore), MAO-A (SC-20156, Santa Cruz, Dallas, TX, USA), and NAO-B (SC-18401, Santa Cruz) were used to detect the protein expression levels and activities. The signals were detected on a ChemiDoc™ XRS+ Image System (Bio-Rad Laboratories, Hercules, CA, USA). The blots were then stripped and reprobed using anti-GAPDH (NB300-221, Novus Biologicals, Littleton, CO, USA), anti-beta-actin (NB-600-501, Novus Biological), or anti-TH (ab137869, Abcam) antibodies for quantitative control analysis.

### 2.8. Statistical and Data Analyses

All data were analyzed using GraphPad Prism software (GraphPad Software Inc., La Jolla, CA, USA). The results are expressed as mean ± SEM. Data were tested using ANOVA with post hoc Bonferroni's multiple comparison.* P* < 0.05 was considered significant.

## 3. Results

### 3.1. Effects of EA on METH-Induced Behavioral Sensitization and CPP

**Figure 2:**
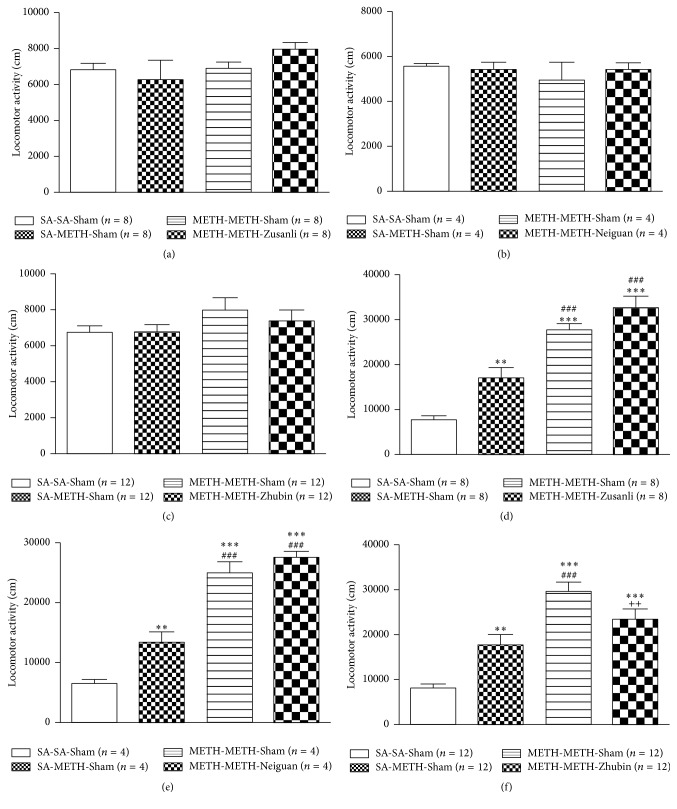
Effects of electroacupuncture intervention at various acupoints on the spontaneous motor activity and methamphetamine- (METH-) induced behavioral sensitization. Mice were given saline or METH (2 mg/kg) intraperitoneally for 5 days. EA was applied from withdrawal days 3 to 5. The behavioral sensitization was induced using 1 mg/kg METH at withdrawal day 6. The bar graphs illustrate the cumulative distances of locomotor activity for a 1 h observation period. Spontaneous motor activity: (a)* Zusanli* (ST36)–*Taichong* (LR3), (b)* Neiguan* (PC6)–*Quchi* (LI11), and (c)* Zhubin* (KI9)–*Taichong* (LR3) and METH-induced behavioral sensitization: (d) ST36–LR3, (e) PC6–LI11, and (f) KI9–LR3. All data are expressed as means ± SEMs (*n* = 4–12 per group). ^*∗∗*^*P* < 0.01 and ^*∗∗∗*^*P* < 0.001 versus the Saline-Saline-Sham group; ^###^*P* < 0.001 versus the Saline-METH-Sham group; ^++^*P* < 0.01 versus METH-METH-Sham group (one-way ANOVA).

**Figure 3:**
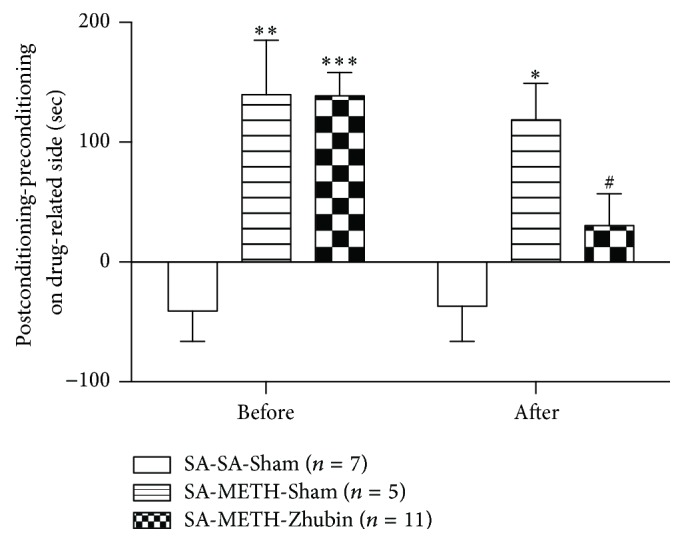
Effects of electroacupuncture (EA) at KI9–LR3 on methamphetamine- (METH-) induced conditioned place preference (CPP). Time scores showing differences before and after conditioning in the saline- or 2 mg/kg METH-paired environment before and after the EA intervention. All data are expressed as means ± SEMs (*n* = 5–11 per group). ^*∗*^*P* < 0.05, ^*∗∗*^*P* < 0.01, and ^*∗∗∗*^*P* < 0.001 versus the Saline-Saline-Sham group; ^#^*P* < 0.05 versus the Saline-METH-Sham group (one-way ANOVA).

After EA application at sham or specific acupoints, basal motor activities did not differ significantly among the groups (Figures 2(a)–2(c)). As depicted in Figure 2, acute METH administration induced locomotor activity and significant behavioral sensitization in the chronic METH-administered group (Figure 2(d), *F*_(3,28)_ = 39.68; Figure 3(b), *F*_(3,12)_ = 45.82; Figure 3(c), *F*_(3,33)_ = 64.39; all* P* < 0.0001). EA at ST36–LR3 (Figure 2(d)) or PC6–LI11 (Figure 2(e)) did not affect METH-induced behavioral sensitization significantly. However, application of EA at KI9–LR3 showed significant decrease in METH-induced behavioral sensitization compared with the EA-Sham group (Figure 2(f), METH-METH-*Zhubin* versus METH-METH-Sham). Furthermore, the CPP test for determining the beneficial effects of METH after EA revealed that EA at KI9–LR3 reduced the METH-induced CPP (Figure 3), thus indicating that EA can reduce METH-induced behavioral changes, possibly with acupoints specificity.

### 3.2. Effects of EA on Dopamine Levels and Turnover Rates in the NAc

**Figure 4:**
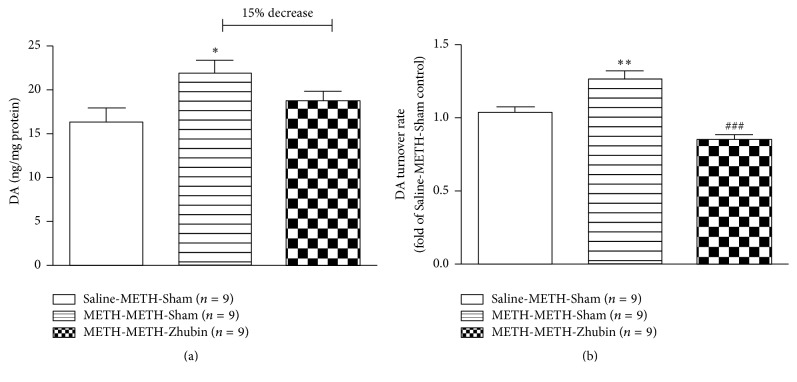
Effects of electroacupuncture at KI9–LR3 on the dopamine levels in the nucleus accumbens (NAc). The levels of dopamine and the related metabolites in the NAc were measured through high-performance liquid chromatography-electrochemical detection after the behavioral test (1 h after 1 mg/kg METH administration): (a) dopamine levels; (b) dopamine turnover rates. All data are expressed as means ± SEMs (*n* = 9 per group). ^*∗*^*P* < 0.05 and ^*∗∗*^*P* < 0.01 versus the Saline-METH-Sham group; ^###^*P* < 0.001 versus the METH-METH-Sham group (one-way ANOVA).

Dopamine levels substantially influence the regulation of psychostimulant-induced behavioral sensitization [4]. For determining whether EA application affected behaviors by regulating the dopamine system, dopamine levels in the NAc were measured 1 h after final METH administration. Dopamine levels were significantly different after acute and chronic METH administrations (Figure 4(a), *F*_(2,24)_ = 3.609,* P* < 0.05, Saline-METH-Sham versus METH-METH-Sham): the chronic METH-Sham group showed higher dopamine levels in the NAc than did the acute METH-Sham group (*P* < 0.05). In the NAc of chronic METH-treated mice, EA at KI9–LR3 for 3 days led to 15% decrease in the reexposure METH-induced increased dopamine levels (METH-METH-*Zhubin* versus METH-SMETH-METH-Sham). Furthermore, the turnover rate of dopamine was also reduced after the EA intervention (Figure 4(b), *F*_(2,24)_ = 23.37,* P* < 0.001): METH reexposure increased the dopamine turnover rate in the NAc of chronic METH-treated mice; however, it decreased after the EA intervention (*P* < 0.001). The results indicate that EA at KI9–LR3 reduces METH-induced behavioral sensitization probably by reducing the increased dopamine levels in the NAc region.

### 3.3. Effects of EA at KI9–LR3 on TH and MAO Isoenzymes Expressions in the NAc

**Figure 5:**
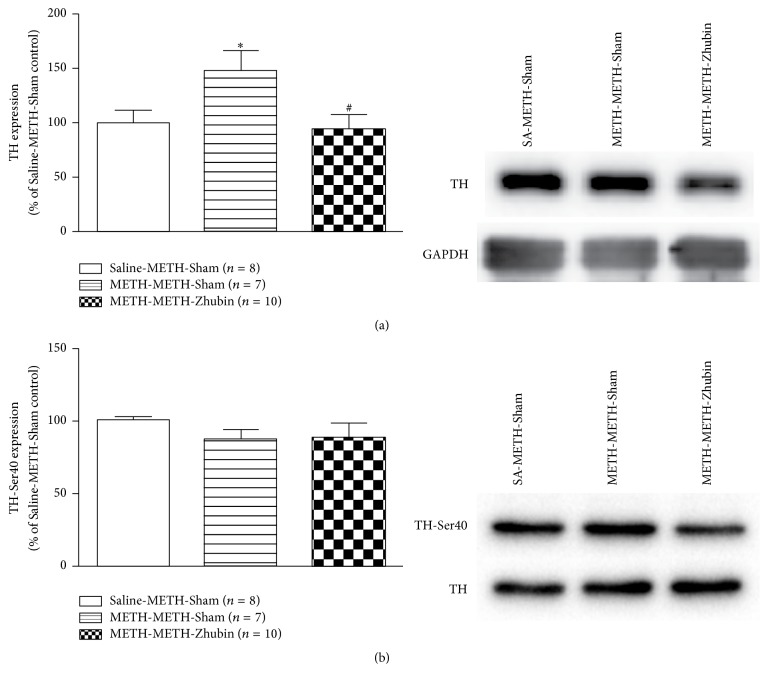
Effects of electroacupuncture on tyrosine hydroxylase (TH) levels in the nucleus accumbens (NAc). TH levels were measured in the NAc after the behavioral test (1 h after 1 mg/kg METH administration). All data are expressed as means ± SEMs (*n* = 7–10 per group). ^*∗*^*P* < 0.05 versus the Saline-METH-Sham group and ^#^*P* < 0.05 versus the METH-METH-Sham group (one-way ANOVA).

**Figure 6:**
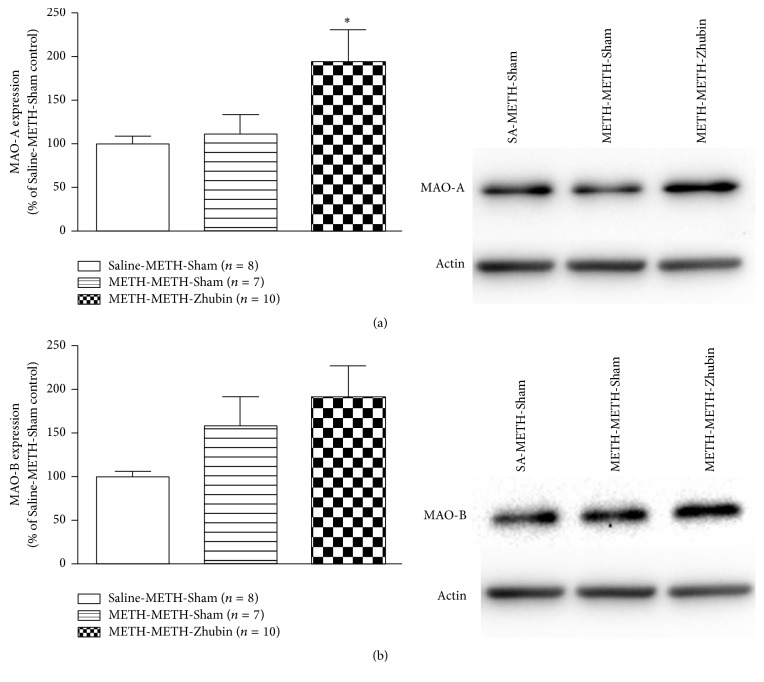
Effects of electroacupuncture on monoamine oxidase isoenzymes A and B (MAO-A/B) levels in the nucleus accumbens (NAc). MAO-A and MAO-B levels were measured in the NAc after the behavioral test (1 h after 1 mg/kg METH administration). All data are expressed as means ± SEMs (*n* = 7–10 per group). ^*∗*^*P* < 0.05 versus the Saline-METH-Sham group (one-way ANOVA).

To determine the possible mechanisms involved in the dopamine level reduction associated with EA, we measured the protein expression of the synthetic enzyme TH in the NAc. As shown in Figure 5(a), TH levels in the NAc of chronic METH-treated mice were significantly reduced after the EA intervention (*F*_(2,22)_ = 3.856,* P* < 0.05). A study suggested that TH phosphorylation can increase its enzyme activity [20]. The regulatory domain of TH contains multiple serine (Ser) residues: its Ser40 site can be phosphorylated by several protein kinases including cAMP-dependent protein kinase, calcium-calmodulin-dependent protein kinase, mitogen-activated-protein-kinase-activating protein kinase, and extracellular-regulated kinase 1/2; the regulatory domain is also associated with the TH activity [20]. Hence, we further investigated whether EA alters the TH activity. The phosphorylation levels of TH-Ser40 were not significantly different after EA intervention at KI9–LR3 between acute and chronic METH-treated mice (Figure 5(b)). In addition, the expression levels of monoamine oxidases were also measured. As shown in Figure 6, the MAO-A expression was significantly increased (*F*_(2,23)_ = 4.039,* P* < 0.05), but no difference was obtained in the MAO-B expression after EA intervention at KI9–LR3. Thus, the EA-related decreases in the dopamine levels may be majorly regulated through TH and MAO-A expression.

## 4. Discussion

This study evaluated the effects of EA on METH-induced behavioral and cellular changes. By using a systemic METH challenge, we induced considerable locomotor activity. The EA intervention during the METH withdrawal period suppressed METH-induced behavioral sensitization. Of the three acupoints pairs, only the KI9–LR3 (but not ST36–LR3 or PC6–LI11) combination reduced the METH-induced increases in the locomotor activity. All acupoints used in the current study are located on the legs; EA on these acupoints can cause muscle trembles, which may disrupt the leg-moving function after repeated stimuli. Nevertheless, according to the results for basal locomotor activities in all tested groups, stimulation of these acupoints did not affect the leg-moving function. Similarly, behavioral sensitization was reduced with EA at KI9–LR3 but not at ST36–LR3 and PC6–LI11. These results suggest that the suppressive effects of EA on METH-induced behavioral sensitization are associated with specific acupoints combinations.

CPP, based on Pavlovian conditioning, is widely used for identifying the addictive liability of compounds in animals [21–23]. EA at KI9–LR3 affected CPP considerably and improved the extinction, indicating that EA at KI9–LR3 may also regulate the brain reward learning system. Dopamine production in the NAc is critical in the brain reward circuitry [4, 24, 25]. Here, dopamine level and turnover rate in the NAc increased significantly after METH administration; nevertheless, both decreased after the EA intervention. Furthermore, dopamine levels are mainly regulated by TH, a crucial regulation factor for METH-induced behavioral and cellular changes [26]. The phosphorylation at Ser40 of TH is associated with TH activity [20, 27]. TH expression decreased after EA at KI9–LR3 in METH-treated mice, whereas EA treatment did not affect the levels of phosphorylation at Ser40 form of TH. Furthermore, the MAO-A expression was significantly increased after EA at KI9–LR3 in METH-treated mice and MAO-A and MAO-B expression also slightly raised after METH administration in this study. MAOs are enzymes which catalyze the oxidative deamination of monoamine neurotransmitters. The degradation of dopamine is mediated by both MAOs, MAO-A has higher affinity for serotonin and norepinephrine, and MAO-B contributes to 2-phenylethylamine and dopamine [28]. But the dichotomy of substrate between MAO-A and MAO-B is not absolute, and it is also related to the species and tissues. In fact, in rodent the dopamine metabolism is prevalently served by MAO-A, while in human and other primates it is MAO-B [28]. And MAOs have a compensation mechanism that when either one is absent, the other can deaminate its nonpreferred monoamine substrate [29]. Although the MAO-A was increased, the turnover rate was also decreased after EA at KI9–LR3, it may be caused by decreasing the TH expression and then reducing the substrate-dopamine levels or a complicated interaction of MAO-A and MAO-B. In addition, the MAO-B expression slightly increased after chronic METH administration, and EA application also caused higher but not significant expression as compared with METH-treated only group. Previously, a gene knockout study suggests that phenylethylamine is majorly metabolized by MAO-B in the rodent [30]. METH might be decomposed into amphetamine by cytochrome P-450s [31] and acute amphetamine administration increased phenylethylamine level in the rodent's brain which was also demonstrated in an early study [32]. Hence, there exists a possible mechanism that chronic METH administration caused increases of amphetamine and subsequently raised phenylethylamine concentration in brain. The higher expression of MAO-B may contribute a homeostasis mechanism to reduce the level of amphetamine-induced phenylethylamine. The results may also imply that EA could reduce phenylethylamine levels via enhancing MAO-B expression in the brain. Unfortunately, there is still a lack of the details of acupuncture regulated MAO expression. Previous studies suggested that acupuncture reduced tumor necrosis factor-*α* (TNF*α*), interleukin-6 (IL-6), C-reactive protein (CRP), and nitric oxide synthase (NOS) in the blood of brain injury rat model [33]. And EA like fluoxetine had an anti-inflammatory effect by reducing interleukin-1 beta (IL-1*β*) to restore the imbalance effects on pro- and anti-inflammatory cytokines in depression patients [34]. Inflammatory cytokines are the important factors for developing and maintaining normal brain functions [35]. Cytokines could regulate the synthesis of dopamine in the basal ganglia [36] and METH treatment also showed increases of TNF*α* and IL-6 expressions in the frontal cortex [37]. Inflammatory effects have also been reported to increase the expression and activity of MAO-A in the neural cells [38, 39]. Based on these findings, EA influences MAO-A expression maybe via regulating the inflammatory cytokines-related pathways. In addition, EA-related reduction in cocaine-induced behavioral sensitization is closely associated with the reduction of dopamine synthesis in the ventral tegmental area (VTA) region [17] and phosphorylated cAMP response element-binding protein expression in the NAc [8]. A study suggested that EA enhances extinction learning of heroin-induced seeking behavior, which may associate with cAMP downstream pathway—FosB expression in the NAc [40]. Our study also revealed similar effects of EA on METH-induced behavioral and cellular changes. Thus, EA may be used as a nonpharmacological therapy for drug addiction by regulating the hemostasis balance mechanism.

A clinical study demonstrated the effectiveness of KI9 in preventing relapse in alcohol-dependent patients [14]; similarly, our preliminary results indicated the usefulness of methadone-induced CPP in mice (data not shown). These findings indicate that only specific acupoints may be useful for treating particular diseases or specific drug-induced behavioral changes. Other studies have also corroborated this argument. Acupuncture treatment at the bilateral HT7* (Shenmen)*, but not PC6* (Neiguan)*, has been shown to reduce cocaine-induced behavioral sensitization and TH expression in the VTA [17] and may be mediated by activation of A*δ* afferent fiber [41]. Similarly, our results suggested that acupuncture at PC6 does not affect METH-induced behavioral sensitization. Because both cocaine and METH are psychostimulants, PC6 may be unsuitable for direct treatment of psychostimulants-induced behavioral changes. Zhao et al. [42] recently suggested that acupuncture at HT7, but not at PC6, reduces alcohol withdrawal effects through increasing TH mRNA and protein expression in the VTA and NAc, respectively, along with dopamine levels in the NAc. However, stimulation of PC6 can effectively treat Internet addiction [43] and alleviate the withdrawal syndrome of heroin addiction [44]; the acupoint is also involved in neurotransmitter regulation [45]. It seems compatible with the above description; ST36 has also been reported to influence opioid-induced addiction and side effects; however ST36 was not effective on the current METH study with no reports of the cocaine studies [46]. All the aforementioned findings suggest that acupuncture at specific acupoints, which regulate the dopaminergic pathway in the NAc, can be used for treating specific drug addictions.

Another factor critically influencing the therapeutic effects of EA may be the frequency of EA intervention. After EA stimulation, several endogenous neuropeptides or neurotransmitters, such as endomorphin, enkephalin, substance P, and serotonin, are released in a frequency-dependent manner [18, 47]. At a lower frequency (2 Hz), EA increases endomorphin, enkephalin, and endorphin release, whereas, at a higher frequency (100 Hz), EA increases dynorphin release [48]. In studies on morphine-induced behavioral changes, 100 Hz EA effectively suppressed the withdrawal syndrome, and CPP could be improved using EA of only 2 Hz [18, 47]. These findings indicate that the frequency of EA is critical for the ideal regulation of neuropeptide and neurotransmitters release.

In traditional Chinese medicine, the clinical functions of KI9 and LR3 are associated with the hepatic and renal detoxification functions, and these points can be used to treat nephritis and hepatitis as well as mental and psychosomatic disorders (such as mania, headache, and insomnia) in humans. Therefore, in this study, KI9–LR3 stimulation may have suppressed METH-induced behavioral changes, possibly through regulation of the metabolism of METH. However, this hypothesis warrants further investigation.

Our results and those in the literature suggest that peripheral stimulation of the acupoints can influence neurotransmitter release and CNS activities [17, 49]. However, the mechanism through which peripheral EA affects CNS functions remains unclear. For example, KI9 is more effective than ST36 in the current study; a possible mechanism may be due to the nerves of acupoints located. According to the acupuncture anatomy [50], KI9 locates on the Tibial nerve, but ST36 locates at a branch of the Common fibular (peroneal) nerve. These two nerves have different afferent pathways to the spinal cord (tibial nerve: L4-S3; Common fibular nerve: L4-S2) and innervate different muscles. The types of neural fibers, such as A*α*, A*β*, A*δ*, and C fiber, have been reported to be involved in EA effects [51]. There may have been different components of neural fibers in the Tibial and Common fibular nerves. However, studies also showed that EA on the same neural fibers with different locations caused different treatment consequences by the different hormone release rates [51]. Hence, the nerves of acupoints located and component of neural fibers in the afferent nerve may play the effective factors for EA application. Furthermore, the molecular changes of acupoints located nerves also play an important role. A recent study demonstrated that the local expression of adenosine and activation of adenosine A1 receptor (A1R) have a critical role in antinociception in the CNS [52, 53]: stimulation of* Zusanli* (ST36) resulted in a considerable, localized, and sustained release of adenosine and adenine nucleotides (AMP, ADP, and ATP) as well as activation of the A1R. This finding is indicative of the association of acupoints with peripheral somatosensory afferents to the CNS, extending its usefulness from acute pain management to chronic pain. Moreover, evidence shows that EA can be used to treat autonomic nerve-related disorder, such as epilepsy, anxiety, analgesia, and nervousness, through the regulation of several brain areas [47, 54]. Drugs of abuse can influence the rewarding system in the brain, such as the prefrontal cortex, NAc, and hippocampus [55]. Acupuncture application can alter cerebral blood oxygenation and modulate prefrontal cortex activity by regulating the autonomic nervous function [56]. Thus, another possible mechanism by which EA influences drug additive liability involves modulation of the afferents of peripheral somatosensory afferents to the CNS.

In traditional Asian medicine, acupuncture is a popular nonpharmacological treatment option for drug addiction, such as that of alcohol, nicotine, and heroin [11, 46]. Acupuncture research is difficult because the accurate treatment duration, acupuncture frequency, duration of stimulation, acupoint and control selection, and sufficient sample size cannot be determined; thus, the effects of acupuncture therapy for drug addiction are controversial, particularly in clinical applications [7, 46, 57, 58]. Nevertheless, the mechanism through which acupuncture alleviates drug addiction probably involves regulation of brain pathways and restoration of the unbalanced central neural pathways through the modulation of neurotransmitters, such as dopamine, norepinephrine, serotonin, and endogenous opioids [46, 47, 59, 60]. Hence, future studies must integrate these basic findings and determine clinical therapeutic effects of EA.

## 5. Conclusion

In summary, our results demonstrated that EA at KI9–LR3 can inhibit repeated METH-induced behavioral sensitization and CPP in mice, possibly by modulating the activity of the dopaminergic system in their NAc. In addition, EA at specific acupoints may be an effective nonpharmacological therapeutic method for METH abuse.

## Abbreviations

A1R: Adenosine A1 receptor
CNS: Central nervous system
CPP: Conditioned place preference
CRP: C-reactive protein
DA: Dopamine
EA: Electroacupuncture
ECD: Electrochemical detector
HPLC: High-performance liquid chromatography
IL-1*β*: Interleukin-1 beta
IL-6: Interleukin-6
MAO: Monoamine oxidase
METH: Methamphetamine
NAc: Nucleus accumbens
TH: Tyrosine hydroxylase
VTA: Ventral tegmental area.
