# Corrigendum to “Effects of Electroacupuncture on Methamphetamine-Induced Behavioral Changes in Mice”

**DOI:** 10.1155/2018/3943120

**Published:** 2018-12-02

**Authors:** Tsung-Jung Ho, Chiang-Wen Lee, Zi-Yun Lu, Hsien-Yuan Lane, Ming-Horng Tsai, Ing-Kang Ho, Chieh-Liang Huang, Yao-Chang Chiang

**Affiliations:** ^1^Center for Drug Abuse and Addiction, China Medical University Hospital, China Medical University, Taichung, Taiwan; ^2^School of Chinese Medicine, China Medical University, Taichung, Taiwan; ^3^Division of Chinese Medicine, China Medical University Beigang Hospital, Yunlin County, Taiwan; ^4^Division of Chinese Medicine, An Nan Hospital, China Medical University, Tainan, Taiwan; ^5^Division of Basic Medical Sciences, Department of Nursing, Chang Gung Institute of Technology and Chronic Diseases and Health Promotion Research Center, Chiayi, Taiwan; ^6^Research Center for Industry of Human Ecology and Research Center for Chinese Herbal Medicine, Chang Gung University of Science and Technology, Taoyuan 33303, Taiwan; ^7^Department of Rehabilitation, Chang Gung Memorial Hospital, Chiayi 61363, Taiwan; ^8^Research Center for Chinese Medicine & Acupuncture, China Medical University, Taichung, Taiwan; ^9^Graduate Institute of Biomedical Sciences and Ph.D. Program for Aging, China Medical University, Taichung, Taiwan; ^10^Department of Psychiatry, China Medical University Hospital, Taichung, Taiwan; ^11^Department of Pediatrics, Division of Neonatology and Pediatric Hematology/Oncology, Chang Gung Memorial Hospital, Yunlin, Taiwan

In the article titled “Effects of Electroacupuncture on Methamphetamine-Induced Behavioral Changes in Mice” [1], there was a missing affiliation for the second author. The corrected authors' list and affiliations are shown above.
